# Biomarkers of food intake for nuts and vegetable oils: an extensive literature search

**DOI:** 10.1186/s12263-019-0628-8

**Published:** 2019-03-19

**Authors:** Mar Garcia-Aloy, Paul J. M. Hulshof, Sheila Estruel-Amades, Maryse C. J. Osté, Maria Lankinen, Johanna M. Geleijnse, Janette de Goede, Marynka Ulaszewska, Fulvio Mattivi, Stephan J. L. Bakker, Ursula Schwab, Cristina Andres-Lacueva

**Affiliations:** 1 Biomarkers and Nutrimetabolomics Laboratory, Department of Nutrition, Food Sciences and Gastronomy, XaRTA, INSA, Faculty of Pharmacy and Food Sciences, Campus Torribera, University of Barcelona, Barcelona, Spain; 2 CIBER de Fragilidad y Envejecimiento Saludable (CIBERFES), Instituto de Salud Carlos III, Barcelona, Spain; 3 Division of Human Nutrition and Health, Wageningen University, Wageningen, the Netherlands; 4 Department of Internal Medicine, University Medical Center Groningen, University of Groningen, Groningen, the Netherlands; 5 School of Medicine, Institute of Public Health and Clinical Nutrition, University of Eastern Finland, Kuopio, Finland; 6 Department of Food Quality and Nutrition, Research Innovation Center, Fondazione Edmund Mach, Via Mach 1, 38010 San Michele all’Adige, TN Italy; 7 Center Agriculture Food Environment, University of Trento, San Michele all’Adige, Italy; 8 Department of Medicine, Endocrinology and Clinical Nutrition, Kuopio University Hospital, Kuopio, Finland

**Keywords:** Nuts, Oils, Biomarker, Intake, Metabolomics

## Abstract

Nuts and vegetable oils are important sources of fat and of a wide variety of micronutrients and phytochemicals. Following their intake, several of their constituents, as well as their derived metabolites, are found in blood circulation and in urine. As a consequence, these could be used to assess the compliance to a dietary intervention or to determine habitual intake of nuts and vegetable oils. However, before these metabolites can be widely used as biomarkers of food intake (BFIs), several characteristics have to be considered, including specificity, dose response, time response, stability, and analytical performance. We have, therefore, conducted an extensive literature search to evaluate current knowledge about potential BFIs of nuts and vegetable oils. Once identified, the strengths and weaknesses of the most promising candidate BFIs have been summarized. Results from selected studies have provided a variety of compounds mainly derived from the fatty fraction of these foods, but also other components and derived metabolites related to their nutritional composition. In particular, α-linolenic acid, urolithins, and 5-hydroxyindole-3-acetic acid seem to be the most plausible candidate BFIs for walnuts, whereas for almonds they could be α-tocopherol and some catechin-derived metabolites. Similarly, several studies have reported a strong association between selenium levels and consumption of Brazil nuts. Intake of vegetable oils has been mainly assessed through the measurement of specific fatty acids in different blood fractions, such as oleic acid for olive oil, α-linolenic acid for flaxseed (linseed) and rapeseed (canola) oils, and linoleic acid for sunflower oil. Additionally, hydroxytyrosol and its metabolites were the most promising distinctive BFIs for (extra) virgin olive oil. However, most of these components lack sufficient specificity to serve as BFIs. Therefore, additional studies are necessary to discover new candidate BFIs, as well as to further evaluate the specificity, sensitivity, dose-response relationships, and reproducibility of these candidate biomarkers and to eventually validate them in other populations. For the discovery of new candidate BFIs, an untargeted metabolomics approach may be the most effective strategy, whereas for increasing the specificity of the evaluation of food consumption, this could be a combination of different metabolites.

**Electronic supplementary material:**

The online version of this article (10.1186/s12263-019-0628-8) contains supplementary material, which is available to authorized users.

## Background

Western diets contain significant but varying amounts of nuts and vegetable oils. Both are natural plant foods rich in fat. Nuts have been a component of the human diet since pre-agricultural times [1]. In Western countries, nuts are consumed either raw or roasted as part of meals, as snacks, or as desserts. They are eaten whole (fresh or roasted), in salads, spreads (in both sweet and salty spreads), as oils or hidden in products, such as sauces, dairies, pastries, and baked goods [2]. Vegetable oils, which can be defined as “oils composed primarily of glycerides of fatty acids being obtained only from plant sources,” have been introduced more recently in Europe. Until the late nineteenth century, the olive was the only edible oil-bearing crop and its use was virtually restricted to the Mediterranean area, while the rest of the continent used animal fats as the principal source of cooking oil [3]. Due to technological developments, large-scale food production, and easier and cheaper transport, the consumption of olive oil and other vegetable oils increased [4].

Nuts are nutrient-dense foods and are rich sources of dietary fatty acids with a high ratio of unsaturated to saturated fatty acids [2]. Moreover, they contain many other nutrients and bioactive compounds, including high-quality proteins, fibers, minerals, tocopherols, phytosterols, and phenolic compounds [2]. The main fatty acids in nuts are oleic acid (C18:1), linoleic acid (C18:2), and α-linoleic acid (C18:3) [5, 6]. Vegetable oils are another important source of dietary fatty acid intake. Globally, the main oils in the human diet are derived from soya, palm, sunflower, and rape [7], although there is high variability depending on the local tradition of each region. These oils are mostly used for baking, frying, or as salad dressing [8]. Vegetable oils are rich sources of (n-9) monounsaturated fatty acids (MUFAs) and (n-6 and n-3) polyunsaturated fatty acids (PUFAs). Hydroxytyrosol [9] is a specific compound associated with olive oil consumption, which is believed to contribute to several of its beneficial health effects [10].

Many studies have investigated the potential health effects of nuts and vegetable oils. Previous epidemiologic studies on the health effects of nuts have shown that nut consumption is associated with a lower incidence of coronary heart disease in both men and women [11]. Additionally, intervention studies have shown an LDL-cholesterol-lowering effect of nut consumption, usually without any effect on HDL-cholesterol and triglycerides [12–14]. Likewise, it is known that isocaloric replacement of saturated fatty acids (SFAs) by MUFAs and PUFAs, which are most common in vegetable oils, is associated with a lower risk of developing cardiovascular diseases, which is partly mediated by lowering LDL-cholesterol [15].

Given the potential health benefits of both nuts and vegetable oils, it is important to find specific biomarkers of their intake. Currently, food frequency questionnaires (FFQs), food diaries, and 24-h dietary recalls are used as dietary assessment tools in studies on nutrition. However, these assessment tools are based on self-reporting by subjects and some of the drawbacks associated with self-reporting food consumption are, among others, that they rely on a correct estimation of portion size. Additionally, surveys based on retrospective methods (such as 24-h dietary recalls or FFQ) depend on the memory of the subject, which could lead to food omissions, while the prospective surveys (such as food diaries) could cause changes in eating behavior. They often focus on type, frequency, and serving size, but do not take into account information on food sources, food processing, or storage conditions. To illustrate, usually the presence of oil in processed foods or receipts is disregarded by consumers, whereas nuts are often hidden in processed foods (for example, in sauces, spreads, dairy products, etc.) and as such these products are easily missed with self-reported dietary assessment methods. Therefore, there is a growing interest in biomarkers of food intake (BFIs), which are a more objective reflection of dietary intake [16]. These biomarker-based measurements of dietary intake are independent of subjects’ memory, misreporting, or limitations of food composition databases and can improve intake measurements, contributing to better estimates of associations between diet and health outcomes. Therefore, the use of BFI as a complementary or alternative tool of the traditional instruments is one of the focus of current and future research topics in nutritional sciences.

This review has been developed as part of the Food Biomarkers Alliance (FoodBAll) consortium, supported by the Joint Programming Initiative “A Healthy Diet for a Healthy Life” [17]. The objective of this paper was to perform an extensive literature search of both observational and human intervention studies in order to describe which BFIs of both nuts and vegetable oils have been described until now.

## Methodology

This review is focused on the most widely consumed types of nuts and vegetable oils. For nuts, walnuts, hazelnuts, pistachios, pecan nuts, macadamia nuts, cashews, and Brazil nuts were selected. Additionally, almonds and peanuts, although they are botanically classified as drupes and legumes, respectively, have also been included because of their nutritional profile. Among vegetable oils, olive, sunflower, flaxseed, and rapeseed oils were covered.

The review was conducted following the methodology harmonized within the FoodBAll consortium (http://foodmetabolome.org/) and recently described [18]. The search was conducted in three databases (PubMed, Scopus, and Web of Science) using the following combinations of grouped search terms: (biomarker* OR marker* OR metabolite* OR biokinetics OR biotransformation) AND (trial OR experiment OR study OR intervention) AND (human* OR men OR women OR patient* OR volunteer* OR participant*) AND (urine OR plasma OR serum OR blood OR excretion OR “adipose tissue” OR “fat tissue” OR “erythrocyte membrane*” OR phospholipid* OR “cholesterol ester*” OR “cholesteryl ester*” OR triglyceride* OR triacylglycerol*) AND (intake OR meal OR diet OR ingestion OR consumption OR eating OR drink* OR administration), together with specific keywords related to each food group, since searches were carried out separately for each food group. For nuts these were (nut OR nuts OR walnut* OR hazelnut* OR almond* OR pecan* OR macadamia* OR peanut* OR pistachio* OR cashew* OR “brazil nut”), whereas for vegetable oils they were (oil*) AND (olive* OR coconut* OR rapeseed* OR canola* OR sunflower* OR palm* OR flaxseed* OR linseed* OR sesame* OR corn* OR soybean* OR safflower* OR seed*). The mentioned keywords were used in the default fields of each database. They were [All fields], [Article Title/Abstract/Keywords], and [Topic] for PubMed, Scopus, and Web of Science, respectively.

Firstly, titles and abstracts were screened to determine whether they met the selection criteria. In case of doubt, the papers were also kept in the list of selected references, which were further evaluated using information included in the full text. Additional papers were identified from reference lists of selected papers and relevant reviews. Only papers in the English language were considered eligible, while no restriction was applied for publication dates (the last search was done in December 2017). Those papers identifying or using potential BFIs of nuts or vegetable oils measured in human biological samples were selected (i.e., animal studies were excluded). Those papers reporting duplicated data from the same study were excluded, with only one paper being retained for each study. The research papers identifying or using potential BFIs were selected by one or more skilled researchers. All candidate BFIs were merged in a unique list, which was further split according to their potentiality as promising candidate BFIs, either used alone (as a single BFI) or within a combination in a multi-metabolite biomarker panel. Those potentially good candidate BFIs were included in a first table together with the description of the corresponding studies where they were measured, while the others were grouped in a second table along with their associated references where the association with the food intake was described, as well as the main reason for exclusion.

Finally, a score system also developed within the FoodBAll consortium [19] was applied for those BFIs retained as potentially good candidates in order to systematically assess their current validity, as well as to pinpoint whether additional studies were still needed. It included eight items related to both analytical and biological aspects.

## Results and discussion

**Fig. 1:**
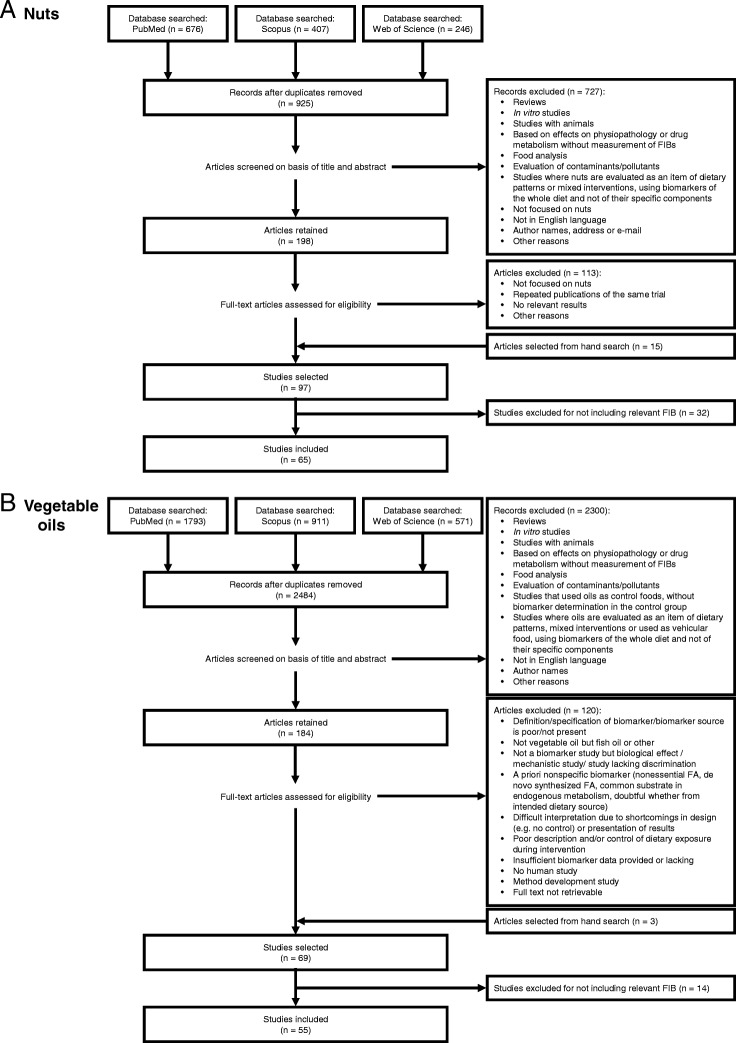
Flow diagram of study selection

The literature has been extensively reviewed independently for nuts and vegetable oils intake biomarkers. Figure 1 presents an overview of the review and study selection process. Firstly, electronic searches were conducted using the Web of Science, PubMed, and Scopus databases. After excluding duplicated references, a total of 925 and 2484 articles were screened for nuts and vegetable oils, respectively. After title and abstract screening, a total of 97 and 69 articles were selected for providing information on potential candidate BFIs of consumption of nuts and vegetable oils, respectively. Further evaluation of the full-text papers reduced the results to 65 and 55 eligible papers to be included in the sections of nuts and vegetable oils, respectively. The results are successively presented below.

### Biomarkers of nut consumption

A description of selected studies reporting associations between nut intake and potentially relevant BFIs is given in Table 1. They are organized according to the types of nuts (walnuts, almonds, hazelnuts, pistachios, Brazil nuts, and mixed nuts), the study design [acute study (i.e., single-dose study), chronic intervention (i.e., follow-up after a continued supplementation for a specific frame of time) or observational study], the types of discriminating metabolites (fatty acids, polyphenol-derived metabolites, etc.) and the publication date. Most of the selected studies were focused on walnuts [12, 20–51], followed by Brazil nuts [52–64], while a lower number of studies were found for almonds [65–72], hazelnuts [73–75], pistachios [76–78], and mixed nuts [79–83]. The initial search also retrieved studies on pecan nuts [84, 85], macadamia nuts [86–88], cashews [89, 90], and peanuts [91–94], but none of them included any potentially relevant BFIs (see Additional file 1: Table S1 for the corresponding reasons). Therefore, they were not included in Table 1. Selected papers presented data from studies with different designs: most of them reported data from nutritional intervention studies, with acute [20–26, 52, 53] or chronic [12, 27–49, 54–62, 65–76, 79–83] intake of nuts, while four of them reported data from observational cohorts [50, 51, 63, 64]. The current available knowledge about different biological and analytical parameters that summarize the potential usefulness of each metabolite as a potential BFI is presented in Table 2, while the information on the food intake biomarkers of nuts considered nonrelevant is presented in Additional file 1: Table S1.

**Table 1:** Studies reporting associations between consumption and potential candidate food intake biomarkers for nuts

Dietary factor [reference]	Study design	Number of subjects	Analytical method	Sample type	Discriminating metabolites/candidate biomarkers
Walnuts^a^ [20]	Acute study	8	GC	Large TAG-rich lipoproteins	α-Linolenic acid
Walnuts^a^ [21]	Acute study	20	GC	LDL cholesteryl esters	α-Linolenic acid
Walnuts [22]	Acute study	40	LC-MS	Urine	Urolithin B glucuronide
Walnuts^a^ [23]	Acute study	16	HPLC	Urine	Urolithin A
Walnuts [24]	Acute study	8	Spectro-photometry	Urine	5-Hydroxyindoleacetic acid
Walnuts [25]	Acute study	3	HPLC	Urine	5-Hydroxyindoleacetic acid
Walnuts [26]	Acute study	31	LC-MS	Serum	5-Hydroxyindoleacetic acid
Walnuts^a^ [12]	Sustained intervention	18	GC	Serum cholesteryl esters	α-Linolenic acid
Walnuts^a^ [27]	Sustained intervention	16	GC	Plasma	α-Linolenic acid
Walnuts^a^ [28]	Sustained intervention	21	GC	Plasma TAG	α-Linolenic acid
Walnuts^a^ [29]	Sustained intervention	55	GC	LDL cholesteryl esters	α-Linolenic acid
Walnuts^a^ [30]	Sustained intervention	18	GC	Plasma	α-Linolenic acid
Walnuts [31]	Sustained intervention	10	TLC	LDL proteins	α-Linolenic acid
Walnuts^a^ [32]	Sustained intervention	40	GC	Serum cholesteryl esters	α-Linolenic acid
Walnuts [33]	Sustained intervention	90	NR	Erythrocytes	α-Linolenic acid
Walnuts^a^ [34]	Sustained intervention	10	GC	Blood drops	α-Linolenic acid
Walnuts [35]	Sustained intervention	39	NR	Erythrocytes	α-Linolenic acid
Walnuts^a^ [36]	Sustained intervention	25	GC	Erythrocytes	Linolenic acid
Walnuts [37]	Sustained intervention	50	GC	Erythrocytes	α-Linolenic acid
Walnuts [38]	Sustained intervention	25	NR	Plasma phospholipids	α-Linolenic acid
Walnuts^a^ [39]	Sustained intervention	21	GC	Erythrocytes	Linolenic acid
Walnuts [40]	Sustained intervention	283	GC	Erythrocytes	α-Linolenic acid
Walnuts^a^ [41]	Sustained intervention	18	GC	Plasma	α-Linolenic acid
Walnuts [42]	Sustained intervention	25	GC-FID	Plasma phospholipids	α-Linolenic acid
Walnuts [43]	Sustained intervention	109	GC-FID	Serum	α-Linolenic acid
Walnuts^a^ [44]	Sustained intervention	45	GC-FID	Erythrocytes	α-Linolenic acid
Walnuts^a^ [45]	Sustained intervention	20	NR	Erythrocytes	α-Linolenic acid
Walnuts^a^ [46]	Sustained intervention	40	GC	Plasma	α-Linolenic acid
Walnuts^a^ [47]	Sustained intervention	63	LC-MS	Plasma / Urine / Prostate gland	Urolithin A glucuronide, urolithin B glucuronide (only in prostate gland)
Walnuts [48]	Sustained intervention	10	LC-MS	Urine / Feces	Urolithin A, urolithin A 3-glucuronide (only urine), isourolithin A, isourolithin A 3-glucuronide (only urine), urolithin B, urolithin B glurcuronide (only urine), urolithin C (only feces)
Walnuts^a^ [49]	Sustained intervention	20	LC-MS	Urine / Feces	Urolithin A, urolithin A glucuronide (only urine), isourolithin A, isourolithin A glucuronide (only urine), urolithin B, urolithin B glucuronide (only urine), urolithin C (only feces)
Walnuts [50]	Observational study	107	LC-MS	Urine	5-Hydroxyindole-3-acetic acid
Walnuts^a^ [51]	Observational study	381	LC-MS	Urine	Urolithin A glucuronide / sulfate / sulfoglucuronide, urolithin B glucuronide, urolithin C sulfate, urolithin C glucuronide, hydroxyindoleacetic acid sulfate
Brazil nuts [52]	Acute study	3	LC-MS	Urine	Selenium
Brazil nuts [53]	Acute study	2	LC-ICP-MS	Urine	Selenium
Brazil nuts [54]	Sustained intervention	15	AAS	Plasma	Selenium
Brazil nuts [55]	Sustained intervention	59	AAS	Plasma	Selenium
Brazil nuts [56]	Sustained intervention	81	AAS	Plasma / Erythrocytes	Selenium
Brazil nuts [57]	Sustained intervention	37	AAS	Plasma / Erythrocytes	Selenium
Brazil nuts [58]	Sustained intervention	91	AAS	Plasma	Selenium
Brazil nuts [59]	Sustained intervention	91	ICP-MS	Plasma	Selenium
Brazil nuts [60]	Sustained intervention	82	AAS	Plasma / Erythrocytes / Urine (24 h) / Hair / Nails	Selenium
Brazil nuts [61]	Sustained intervention	31	AAS	Plasma	Selenium
Brazil nuts [62]	Sustained intervention	32	NS	Plasma	Selenium
Brazil nuts [63]	Observational study	155	ICP-MS	Blood	Selenium
Brazil nuts [64]	Observational study	219	ICP-MS	Whole-blood	Selenium
Almonds [65]	Sustained intervention	16	HPLC	Plasma / Erythrocytes	α-Tocopherol
Almonds [66]	Sustained intervention	20	HPLC	Plasma	α-Tocopherol
Almonds [67]	Sustained intervention	60	HPLC	Serum	α-Tocopherol
Almonds [68]	Sustained intervention	24	NR	Plasma	α-Tocopherol
Almonds [69]	Sustained intervention	65	HPLC-FLD	Plasma	α-Tocopherol
Almonds [70]	Sustained intervention	22	HPLC	Plasma	α-Tocopherol
Almonds [71]	Sustained intervention	60	HPLC	Plasma	α-Tocopherol
Almonds [72]	Sustained intervention	45	HPLC	Plasma	α-Tocopherol
Hazelnuts [73]	Sustained intervention	48	HPLC	Plasma	α-Tocopherol
Hazelnuts^a^ [74]	Sustained intervention	21	HPLC	Serum / Isolated LDL	α-Tocopherol
Hazelnuts [75]	Sustained intervention	72	HPLC	Plasma	α-Tocopherol
Pistachios [77]	Sustained intervention	28	GC	Serum	β-Sitosterol
Pistachios^a^ [78]	Sustained intervention	28	HPLC	Serum	Lutein
Pistachios^a^ [76]	Sustained intervention	54	LC-MS	Plasma	Lutein-zeaxanthin
Mixed nuts (walnuts, almonds and hazelnuts) [79]	Sustained intervention	27	GC	Plasma	α-Linolenic acid
Mixed nuts (walnuts, almonds and hazelnuts) [80]	Sustained intervention	375	GC	Plasma	α-Linolenic acid
Mixed nuts (walnuts, almonds and hazelnuts) [81]	Sustained intervention	42	LC-MS	Urine	Urolithin A glucuronide / sulfate / sulfoglucuronide, hydroxyindoleacetic acid
Mixed nuts (walnuts, almonds and hazelnuts) [82]	Sustained intervention	41	LC-MS	Urine	Urolithin A, urolithin B
Mixed nuts (walnuts, almonds and hazelnuts) ^a^ [83]	Sustained intervention	47	LC-MS	Plasma	Urolithin A glucuronide

*AAS* atomic absorption spectrometry, *FID* flame ionization detector, *FLD* fluorometric detection, *GC* gas chromatography, *HPLC* high-performance liquid chromatography, *ICP* inductively coupled plasma, *MS* mass spectrometry, *NR* not reported, *TAG* triacylglycerides, *TLC* thin-layer chromatography

^a^The study includes other nonrelevant metabolites (included in Additional file 1: Table S1)

**Table 2:** Validation scheme of potential food intake biomarkers for nuts

Dietary factor [references]	Compound/metabolite	HMDB ID	Sample type	Criteria								
				1	2	3a	3b	4	5	6	7	8
Walnuts [12, 20, 21, 28–45, 27, 46, 79, 80]	α-Linolenic acid	HMDB0001388	Plasma / serum / erythrocytes	N	U	U	U	U	U	U	Y	U
Walnuts [22, 23, 48, 47, 49, 51, 82, 81, 83]	Urolithins: urolithin A (and phase II metabolites), isourolithin A (and phase II metabolites), urolithin B (and phase II metabolites), urolithin C (and phase II metabolites)	HMDB0013695 / HMDB0029222 / HMDB0060022 / HMDB0013696 / HMDB0041787 / HMDB0029218	Urine / plasma	N	U	Y	U	Y	U	U	Y	U
Walnuts [24, 50, 51, 81]	5-Hydroxyindole-3-acetic acid	HMDB0000763	Urine / serum	N	Y	Y	U	Y	U	U	Y	U
Almonds/hazelnuts [66–72]	α-Tocopherol	HMDB0001893	Plasma / serum / erythrocytes	N	U	U	U	U	U	U	Y	U
Pistachios [77]	β-Sitosterol	HMDB0000852	Serum	N	U	U	U	U	U	U	Y	U
Pistachios [76, 78]	Lutein-zeaxanthin	HMDB0003233/HMDB0002789	Plasma / serum	N	U	U	U	U	U	U	Y	U
Brazil nuts [53–61]	Selenium	HMDB0001349	Urine / plasma	N	Y	Y	Y	Y	U	U	Y	U

*HMDB* human metabolome database, *N* no, *U* unknown, *Y* yes. Criteria: *C1*—*Plausibility*, Is the marker compound plausible as a specific BFI for the food or food group?; *C2*—*Dose response*, Is there a dose-response relationship at relevant intake levels of the targeted food?; *C3*—*Time response*, Is the biomarker kinetics described adequately to make a wise choice of sample type, frequency and time window?; *C3a*, *single dose*; *C3b*, *multiple doses*; *C4*, *Robustness*, Has the marker been shown to be robust after intake of complex meals reflecting dietary habits of the targeted population?; *C5*, *Reliability*, Has the marker been shown to compare well with other markers or questionnaire data for the same food/food group?; *C6*, *Stabilit*y, Is the marker chemically and biologically stable during biospecimen collection and storage, making measurements reliable and feasible?; *C7*, *Analytical performance*, Are analytical variability (CV%), accuracy, sensitivity and specificity known as adequate for at least one reported analytical method?; *C8*, *Reproducibility*, Has the analysis been successfully reproduced in another laboratory?

Although most of the studies applied targeted approaches, the search strategy also retrieved some untargeted studies. Their inclusion or not in the present review was done based on the potentiality of the reported BFI, regardless of the analytical approach used. Therefore, some of the selected papers that used an untargeted strategy were retained as being particularly interesting because they discovered potentially relevant BFIs of nuts, whereas others were not further considered because they did not report any specific BFI. They were focused on walnuts [50, 51], almonds [95], pistachios [96], peanuts [97], and mixed nuts [81, 83, 98, 99]. Some of these studies reported results similar to the targeted approaches, confirming the relationships between walnut intake and urolithins, fatty acids and serotonin-derived metabolites [50, 51, 81, 83], and almond intake and catechin-derived metabolites [95] (see the corresponding subsections for more detailed information). Neither the latter study on catechin-derived metabolites nor targeted studies reporting results in the same direction [100–102] were retained among the studies reporting relevant candidate BFIs. This was because catechin-derived metabolites have broadly been reported to increase after the intake of other flavan-3-ol-rich food sources, including tea, cocoa, and red wine [103]. Guertin et al. (2014) [97] analyzed the correlations between serum metabolic profiles and peanut consumption according to data from FFQs in participants from the Prostate, Lung, Colorectal, and Ovarian Cancer Screening Trial (PLCO). With this approach, tryptophan betaine and 4-vinylphenol sulfate were proposed as candidate biomarkers of peanut intake. Both metabolites were also associated with nut intake in a case-control study [99]. Tryptophan betaine is an indole alkaloid previously also associated with peanut consumption after being detected in the breast milk of breastfeeding mothers [92]. However, it is also detected in legumes [104–106]. 4-vinylphenol is a catabolite generated by the direct decarboxylation of p-coumaric acid [107]. It would be interesting to go into greater depth with these metabolites in order to work out whether they could be considered as potential BFIs of peanuts. However, with the current knowledge, they could not be included in the list of the most promising candidate BFIs due to potential low specificity.

#### Walnuts

Nuts in general are a rich source of dietary fatty acids with a high unsaturated-to-saturated ratio. The main fatty acids in nuts are oleic acid (C18:1), linoleic acid (C18:2), and α-linolenic acid (C18:3, ALA). Walnuts are characterized by considerably higher amounts of ALA than other types of nuts (11.6% of total fatty acid composition for walnuts compared to < 0.7% for the others) [5, 6]. Such a composition explains the fact that among the different types of nuts, only walnut intake has been associated with ALA in blood both in studies only focused on consumption of walnuts [12, 20, 21, 27–46], and in studies with mixed nut intake that included walnuts [79, 80]. Linoleic acid (C18:2, LA) is the major PUFA present in most types of nuts (40–60% of total fatty acid composition for walnuts, pecans, peanuts, and Brazil nuts) [5, 6]. Therefore, it was consistently found in blood after walnut intake [12, 27–30, 32, 36, 39, 41, 45, 46, 108], and in studies with mixed nuts that included walnuts in their composition [109, 110]. Additionally, its presence in biological fluids was also associated with consumption of cashews [89], for which it is the second most abundant type of fatty acid (20.8%) [6]. Looking at the above-mentioned studies, ALA seems a better candidate biomarker of walnut intake than LA. Nevertheless, there are other food sources of ALA and LA, such as vegetable oils (flaxseed, linseed and rapeseed oils for ALA, and safflower, sunflower, soybean, and corn oil for LA), seeds, and animal products (see the section below dedicated to vegetable oils). This clearly means that the presence of neither ALA nor LA in biological fluids can solely indicate intake of nuts or walnuts. Additionally, both ALA and LA undergo biotransformations in the human body to longer-chain fatty acids [111], giving rise to eicosapentaenoic acid (C20:5, EPA) and docosahexaenoic acid (C22:6, DHA), respectively. Indeed, both of them have been reported after the intake of walnuts [27, 34, 109]. Also, in this case, a confounding factor may occur, as EPA and DHA are also related to fish consumption [112].

Oleic acid (C18:1) is the major MUFA present in most types of nuts (walnuts, almonds, peanuts, hazelnuts, macadamia nuts, and pecan nuts [5, 6]). As a consequence, higher amounts of this fatty acid have been observed in blood and urine after the intake of walnuts [28], almonds [113], hazelnuts [74, 114], pecan nuts [85], macadamia nuts [88], cashews [89, 90], and mixed nuts [98, 115]. This common presence in many types of nuts excludes oleic acid as a direct link to specific nut intake. Moreover, oleic acid has also been associated with olive oil intake (see the corresponding section below). In some targeted investigations, myristic acid (14:0) [12] and stearic acid (18:0) [44, 46], which are the major saturated fatty acids (SFAs) in walnuts, were reported in biological fluids after walnut intake [5]. However, myristic acid is also abundant in dairy products and has been proposed as a potential biomarker of dairy fat intake [116]. In summary, among the different types of fatty acids in walnuts, ALA is the most suitable candidate BFI for walnuts, although it is not specific for this food. For this reason, it seems necessary to perform a complementary search for other potential BFIs of walnuts that are not detected after the consumption of the other ALA food sources [117]. Importantly, McKay et al. [39] analyzed the percentage change in ALA levels compared to baseline levels following an ingestion of 21 g/day or 42 g/day of walnuts for 6 weeks. Although the magnitude of changes in ALA levels after 6 weeks seemed to be higher with the 42 g/day dose (which was the only dose that reached statistical significance compared to baseline), the authors did not make any reference to the potential differences (or not) between the two doses. Therefore, the dose-response association between walnut consumption and ALA levels needs to be further explored. Also, the time-response relationship needs to be further investigated, since neither of the available acute studies reporting levels of ALA after walnut consumption provided a kinetics description [20, 21], but rather they only provided data on one specific time point after consumption. Although the results of the present review did not find any observational study reporting positive associations between levels of ALA and walnut intake, the participants in the study of McKay et al. [39] were not instructed to limit the consumption of other n-3 fatty-rich foods (including fatty fish), thereby reflecting the robustness of this potential BFI in the general population, regardless of the background diet. As regards the analytical performance, various quantification methods using gas chromatography platforms have been developed [118, 119]. However, we could not find any report regarding the reliability (comparison with other BFIs or reference methods), stability during sample collection, storage and processing, or interlaboratory variation.

The appearance in biofluids of urolithins has been the subject of investigations by several authors. In terms of nuts, they have only been reported after intake of walnuts [22, 23, 47–49, 51] or mixed nuts including walnuts [81–83]. In most of these studies, the aglycone or phase II metabolites of urolithin A and B were the most frequently reported metabolites. Urolithins are the product of polymeric ellagitannins (ETs) metabolized by gut microbiota. Among different types of nuts, they are specific for walnuts, but they have also been reported after the intake of pomegranate, strawberries, raspberries, and blackberries. However, these additional foods do not provide important amounts of fatty acids. Therefore, through the employment of a multi-metabolite model, the presence of urolithins and fatty acids at the same time could reveal walnut intake with higher specificity [117]. With regard to the dose-response associations, although there are no studies with different doses of walnuts, one of the selected studies provided participants with different doses of ETs [22]. In that investigation, the subjects consumed different ET amounts through the intake of raspberries (422 mg of ellagic acid, EA), walnuts (191 mg of EA), strawberries (190 mg of EA), or red wine (5.4 mg of EA). The mean highest excretion of urolithins was observed in the walnut group and the lowest in the red wine group. Therefore, the excretion was not directly proportional to the amount of ETs consumed. Instead, it seems that the food matrix has an impact on the bioavailability and metabolism of ETs, which is expected since they exhibit a considerable structural diversity according to food source (i.e., pedunculagin is the major ET found in walnuts; while punicalagins and punicalins predominate in pomegranates; sanguiin H6, sanguiin H10, and lambertiancin C are the main ETs found in berries) [120]. Also, in this study, researchers detected these metabolites in samples collected 16 h after intake, whereas only trace amounts were detected in samples collected before this time point. The complete clearance of ET metabolism could not be estimated since these metabolites were still detected during the following 40 h, when the last sample was collected [22]. Urolithins have also been shown to be a discriminant of walnut consumption in observational studies [51], also highlighting their robustness as BFIs of walnuts in free-living conditions without dietary restrictions, and demonstrating that their levels from potential confounding foods are low. Also in this case, analytical methods have been reported for the quantification of these metabolites in biological samples [121], but we could not find any information related to their stability or interlaboratory reproducibility.

Finally, walnut consumption has also been associated with an increase in the levels of 5-hydroxyindole-3-acetic acid (5-HIAA) [24–26, 50, 51, 81], which is a metabolite of the serotonin pathway. Walnuts have a higher serotonin content than other foods [24], and 5-HIAA has been described as a discriminant metabolite of walnut consumption in two independent observational studies [50, 51], which reinforces its plausibility as a robust BFI for walnuts. Feldman and Lee [24] reported a dose-dependent relationship between the ingested amount of walnuts and the urinary 5-HIAA excretion: 16 units of walnut consumption caused an excretion of 26.0 mg of 5-HIAA in 24 h in urine, while double the amount of walnuts caused excretion of 59 mg/24 h of 5-HIAA. A parallel observation was made when serotonin was provided by other food sources [25]. Additionally, in a more recent study, the authors also used different serotonin food sources [26]. However, the serum levels of 5-HIAA were higher in samples from subjects who consumed the richest source of serotonin (i.e., walnuts) in an amount proportional to the amount provided by each food source. It has been demonstrated that the levels of this metabolite increase within 2 h after the consumption of serotonin-containing foods, and from that moment the concentrations start to decrease, reaching the baseline values within 24 h [25, 26]. Again, analytical methods for the quantification of this metabolite in biological samples have been published [26], but we could not find any data about its stability during sample collection, storage and processing, or interlaboratory reproducibility. However, it has also been reported after intake of other foods such as bananas [24]. Although the contents of serotonin are much higher in walnuts (> 50 μg/g) than in these other potential sources (for instance, bananas contain about 15 μg/g) [24], it is important to consider also the size of a typical serving, since it will influence in the final absolute consumption. For example, the ingestion of serotonin through a typical dose of 30 g walnuts is approximately the same than the one obtained by the consumption of an average-sized banana of 120 g. Furthermore, 5-HIAA has also been reported after the consumption of a Jerte Valley cherry product [122]. The concentration of serotonin in other common nuts like almonds is low (≤ 0.6 μ/g) [24].

**Fig. 2:**
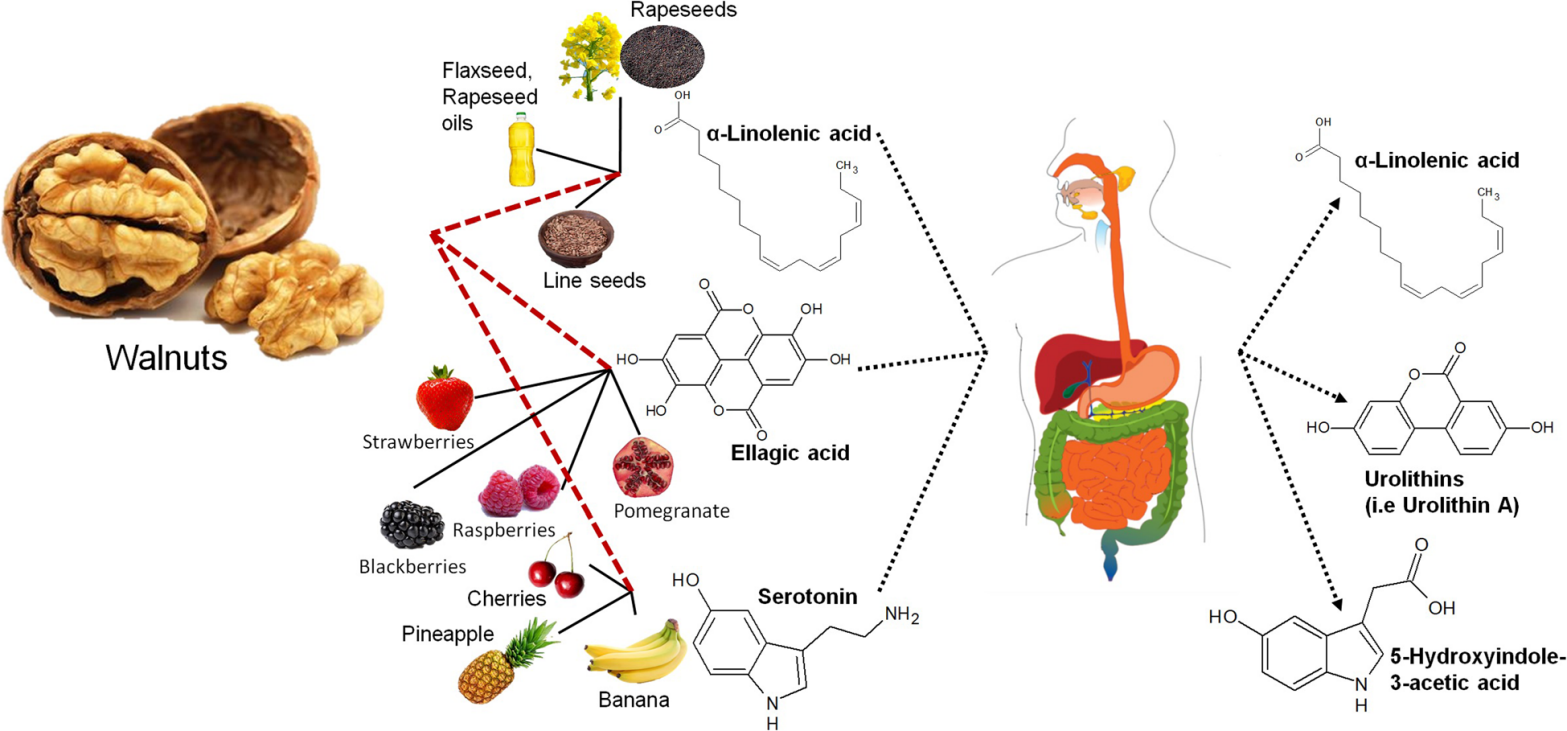
Schematic diagram of combining medium-specific single biomarkers to create a more specific multi-metabolite biomarker panel

Therefore, as already highlighted in the previous paragraph, this is a clear example where multi-metabolite biomarker models may help to overcome the challenge of having a specific measurement [117]. This concept is outlined in Fig. 2, where it is schematized that although none of the candidates as BFIs for walnuts are highly specific when used as a single BFI (the most frequently used approach until now), the panel of them might be characteristic of no common food source other than walnuts.

#### Almonds and hazelnuts

Another important group of nuts revised in this paper are almonds and hazelnuts, which are associated with increased levels of α-tocopherol [65–75]. Almonds and hazelnuts, respectively, have the highest concentrations of α-tocopherol among nuts [5, 6]. However, seeds and vegetable oils, green leafy vegetables, fortified cereals, and tomatoes are also important dietary sources of α-tocopherol [65].

Additionally, flavan-3-ol-derived metabolites have also been associated with almond consumption, although they are also characteristic for tea, wine, and cocoa intake [103]. Therefore, joint measurements of α-tocopherol and flavan-3-ol-derived metabolites could be used to obtain more specific information about almond intake. Nevertheless, additional untargeted metabolomics studies would be useful for proposing complementary metabolites to build multi-metabolite biomarker panels [117].

#### Pistachios

Among nuts, pistachios contain the highest levels of potassium, γ-tocopherol, vitamin K, phytosterols (mainly β-sitosterol), and xanthophyll carotenoids (lutein and zeaxanthin). The number of studies considering pistachio consumption and further metabolite measurements in biological fluids is very limited [76–78]. Lutein and zeaxanthin are particularly interesting compounds as they are very characteristic of pistachios, among other nuts, although they are also frequently present in a wide range of fruits, vegetables [in particular maize (corn) and green leafy vegetables such as spinach], and egg yolk [123, 124]. Two studies included a targeted quantitative analysis of these compounds in plasma after a dietary intervention with pistachios [76, 78]. An investigation by Hernandez-Alonso et al. [76] focused on the relationship between pistachio consumption and the improvement of cardiometabolic risk markers. In this crossover clinical trial, lutein and zeaxanthin, together with α-tocopherol, were proposed as indicators of pistachio intake to monitor the compliance with the dietary intervention. Volunteers were assigned to control diet or a pistachio-supplemented diet (57 g/day) for 4 months. These compounds were measured in fasting plasma at baseline, after a 2-week run-in period and then monthly until the end of each intervention period, and were shown to be statistically significant in the pistachio-supplemented group. However, different results were reported in a crossover, dose-response study performed by Kay et al. [78]. In this case, the researchers only found significant increases of lutein in serum after adding one or two daily servings of pistachios to their diets, whereas no changes from baseline levels in the concentrations of either zeaxanthin or α-tocopherol were observed.

In a study by Holligan et al. (2014), β-sitosterol in plasma was used to verify compliance with the diet (control diet vs diet with one serving of pistachios vs diet with two servings of pistachios for 4 weeks) [77]. The levels of β-sitosterol increased dose dependently and were found to be consistent with dietary approximations from daily questionnaires.

In summary, the above-mentioned reported investigations used the measurement of lutein, zeaxanthin, β-sitosterol, and α-tocopherols (pistachio components) to verify compliance with diets rich in pistachios. All of these compounds are common for many fruits and vegetables, as well as for other types of nuts, and thus cannot be considered specific metabolites of pistachio intake. Only one study was found that used an untargeted metabolomics approach to study the metabolic response in biological fluids after pistachio consumption [125]. However, it could not be included in the present review because it only reported on changes in endogenous metabolites. Therefore, additional complementary human trials with the use of untargeted metabolomics might reveal additional compounds or metabolites that could be suggested as potential biomarkers of intake.

#### Brazil nuts

Brazil nuts are one of the food sources with the highest contents of selenium. Accordingly, high levels of selenium have been reported in several studies after intake of Brazil nuts [52–64]. Although this essential mineral is found in many foods, the most relevant dietary source of selenium is Brazil nuts. However, it is important to keep in mind that it is also used in dietary supplements or in enriched foods, as well as that different geographical factors, such as selenium concentration in the soil (which varies from region to region), impact on the selenium content [126]. Selenium has also been observed to be a discriminant of Brazil nut consumption, independently of the background diet [63, 64]. The highest urinary selenium concentrations have been measured 4 h after consumption of Brazil nuts and even higher concentrations have been observed after repeated intakes [52]. Therefore, it remains to be clarified whether the use of only this compound is enough to measure the consumption of Brazil nuts or whether other complementary metabolites should be used jointly for reliable intake assessment.

### Biomarkers of intake of vegetable oils

Biomarkers of vegetable oil intake have been studied most often by linking the intake of fatty acids from these oils to blood plasma and cell responses using controlled intervention studies [127–134]. The main oils studied were olive oil [127–158], flaxseed oil [159–173], rapeseed (canola) oil [157, 158, 174–179], and sunflower oil [157, 173, 178–180]. The study designs include acute studies [133–138, 140, 181], and parallel and crossover dietary intervention studies that vary in the level of control [127–132, 141–180]. These studies were often driven by examining the effects of fatty acids on cardiovascular risk factors such as changes in lipoproteins and hemodynamic factors in low- and high-risk subjects, thereby measuring compliance with dietary exposure. The biological specimens analyzed included plasma and plasma lipid fractions, such as cholesteryl esters and phospholipids, blood platelets, erythrocytes, and adipose tissue. In the case of (virgin) olive oil, the excretion of ingested polyphenols and their metabolites in urine and plasma was also studied. The information in regard to selected studies reporting associations between the consumption of vegetable oils and potential relevant BFIs is summarized in Table 3, while the information regarding the putative BFIs for vegetable oils is given in Table 4 and the information concerning the potential BFIs of vegetable oils that were considered nonrelevant is given in Additional file 1: Table S2.

**Table 3:** Studies reporting associations between consumption and potential candidate food intake biomarkers for vegetable oils

Dietary factor [reference]	Study design	Number of subjects	Analytical method	Sample type	Discriminating metabolites/candidate biomarkers
Olive oil [127]	Sustained intervention	11	GC-FID	Erythrocytes	Oleic acid
Olive oil [128]	Sustained intervention	12	GC-FID	Plasma	Oleic acid
Olive oil^a^ [129]	Sustained intervention	30	GC-FID	Plasma	Oleic acid
Olive oil [130]	Sustained intervention	21	GC-FID	Plasma/platelets	Oleic acid
Olive oil [131]	Sustained intervention	16	GC-FID	Erythrocytes	Oleic acid
Olive oil	Sustained intervention	30	GC-FID	Plasma/PBMC	Oleic acid
Olive oil, extra virgin [133]	Acute study	10	GC	Plasma—TAG	Oleic acid
Olive oil, pomace and refined [134]	Acute study	10	GC	Serum—TRL	Oleic acid
Olive oil, virgin^a^ [135]	Acute study	11	UPLC-MS; GC-MS	Urine	Hydroxytyrosol
Olive oil, different phenolic content^a^ [136]	Acute study	12	GC-MS	Urine	Hydroxytyrosol, 3-O-methy-hydroxytyrosol
Olive oil, enriched or virgin^a^ [137]	Acute study	13	UPLC-MS/MS	Plasma	Hydroxytyrosol sulfate
Olive oil, virgin [138]	Acute study	13	UPLC-MS/MS	Plasma	Hydroxytyrosol sulfate
Olive oil, high phenolic content [139]	Acute study	12	UPLC-MS/MS	Plasma	Hydroxytyrosol sulfate, hydroxytyrosol acetate sulfate
Olive oil, virgin, moderate and high phenolic content [140]	Acute study	13	UPLC-MS/MS	Plasma	Hydroxytyrosol sulfate, hydroxytyrosol acetate sulfate
Olive oil, extra virgin [141]	Sustained intervention	10	GC-FID	Plasma and cells	Oleic acid
Olive oil, extra virgin [142]	Sustained intervention	424	GC	Plasma	Oleic acid
Olive oil, different phenolic^a^ compound content [143]	Sustained intervention	30	GC-MS	Urine	Hydroxytyrosol
Olive oil, different phenol content^a^ [144]	Sustained intervention	200	GC-MS	Urine	Hydroxytyrosol
Olive oil, high-phenol vs low-phenol extra virgin^a^ [145]	Sustained intervention	10	GC-MS	Urine	Hydroxytyrosol
Olive oils, different phenolic content^a^ [146]	Sustained intervention	30	HPLC	Urine	Hydroxytyrosol
Olive oil, different phenolic content^a^ [147]	Sustained intervention	38	GC-MS	Urine	Hydroxytyrosol
Olive oil, refined, common and virgin^a^ [148]	Sustained intervention	33	HPLC	Urine	Hydroxytyrosol
Olive oil, extra virgin^a^ [149]	Sustained intervention	20	HPLC-MS	Plasma	Hydroxytyrosol
Olive oil, extra virgin [80]	Sustained intervention	750	GC-MS	Urine	Hydroxytyrosol
Olive oil, different phenolic compound content^a^ [150]	Sustained intervention	28	GC-MS	Urine	Hydroxytyrosol, O-methylhydroxytyrosol
Olive oil, with different phenolic contents^a^ [151]	Sustained intervention	182	GC-MS/MS	Urine	Hydroxytyrosol, 3-O-methylhydroxytyrosol
Olive oil, virgin and refined^a^ [152]	Sustained intervention	36	HPLC-MS/MS	Plasma-LDL	Hydroxytyrosol sulfate
Olive oil, virgin, enriched [153]	Sustained intervention	33	UHPLC-MS/MS	Urine	Hydroxytyrosol sulfate
Olive oil, high polyphenol content^a^ [154]	Sustained intervention	51	UHPLC-MS/MS	Plasma-HDL	Hydroxytyrosol sulfate
Olive oil, virgin, high phenolic^a^ [155]	Sustained intervention	5	UPLC-MS/MS	Urine	Hydroxytyrosol sulfate, hydroxytyrosol acetate sulfate
Olive oil, virgin, enriched [156]	Sustained intervention	33	UPLC-MS/MS	Plasma/urine	Hydroxytyrosol sulfate, hydroxytyrosol acetate sulfate
Olive oil, virgin (OO); rapeseed oil (RO); sunflower oil (SO) [157]	Sustained intervention	18	GC-FID	Plasma	Oleic acid (OO, RO), linoleic acid (SO, RO)
Olive oil (OO), canola oil (CO) ^a^ [158]	Sustained intervention	14	GC-FID	Plasma	Oleic acid (OO, α-Linolenic acid (CO)
Flaxseed oil [159]	Sustained intervention	16	GC-FID	Plasma/PBMC	α-Linolenic acid
Flaxseed oil [160]	Sustained intervention	30	GC-FID	Plasma	α-Linolenic acid
Flaxseed oil [161]	Sustained intervention	46	GC-FID	Platelets	α-Linolenic acid
Flaxseed oil [162]	Sustained intervention	28	GC-FID	Plasma	α-Linolenic acid
Flaxseed oil [163]	Sustained intervention	17	GC-FID	Plasma/platelets	α-Linolenic acid
Flaxseed oil [164]	Sustained intervention	20	GC	Plasma/erythrocytes	α-Linolenic acid
Flaxseed oil [165]	Sustained intervention	51	GC	Erythrocytes	α-Linolenic acid
Flaxseed oil [166]	Sustained intervention	62	GLC	Erythrocytes	α-Linolenic acid
Flaxseed oil [167]	Sustained intervention	86	GC	Plasma	α-Linolenic acid
Flaxseed oil [168]	Sustained intervention	34	GC	Plasma	α-Linolenic acid
Flaxseed oil [169]	Sustained intervention	98	GC	Erythrocytes	α-Linolenic acid
Flaxseed oil [170]	Sustained intervention	26	GC-FID	Plasma	α-Linolenic acid
Flaxseed oil [171]	Sustained intervention	37	GC	Erythrocytes	α-Linolenic acid
Flaxseed oil [172]	Sustained intervention	15	GC-FID	Serum	α-Linolenic acid
Flaxseed oil (FO); sunflower oil (SO) [173]	Sustained intervention	10	GC-FID	Platelets	α-Linolenic acid (FO); linoleic acid (SO)
Rapeseed oil [177]	Sustained intervention	40	GC-FID	Plasma	α-Linolenic acid
Canola oil^a^ [174]	Sustained intervention	14	GC-FID	Breastmilk	α-Linolenic acid
Canola oil [175]	Sustained intervention	130	GC-FID	Plasma	α-Linolenic acid
Canola oil [176]	Sustained intervention	130	GC-FID	Plasma	α-Linolenic acid
Canola oil (CO); sunflower oil (SO) ^a^ [178]	Sustained intervention	8	GC-FID	Plasma	α-Linolenic acid (CO); linoleic acid (SO)
Canola oil (CO); sunflower oil (SO) [179]	Sustained intervention	8	GC-FID	Platelets	α-Linolenic acid (CO); linoleic acid (SO)
Sunflower oil [180]	Sustained intervention	37	GC	Plasma-CE/ASAT	Linoleic acid

*ASAT* abdominal subcutaneous adipose tissue, *CE* cholesterol esters, *FID* flame ionization detector, *GC* gas chromatography, *GLC* gas-liquid chromatography, *HPLC* high-performance liquid chromatography, *MS* mass spectrometry, *MS*/*MS* tandem mass spectrometry, *PBMC* peripheral blood mononuclear cell, *TAG* triacylglycerides, *UHPLC* ultra-high performance liquid chromatography

^a^The study includes other nonrelevant metabolites (included in Additional file 1: Table S2)

**Table 4:** Validation scheme of potential food intake biomarkers for vegetable oils

Dietary factor [references]	Compound/metabolite	HMDB ID	Sample type	Criteria								
				1	2	3a	3b	4	5	6	7	8
Olive oil [128, 133, 134, 141, 142, 157, 158]	Oleic acid	HMDB0000207	Plasma/blood cells	N	U	Y	Y	Y	U	U	Y	U
Olive oil, (extra) virgin [80, 135, 136, 144–150, 143, 151]	Hydroxytyrosol	HMDB0005784	Urine/plasma	Y	Y	Y	U	Y	U	U	Y	U
Olive oil, (extra) virgin [137, 138, 153–155, 152, 156]	Hydroxytyrosol sulfate	–	Urine/plasma	Y	Y	Y	U	Y	Y	U	Y	U
Olive oil, (extra) virgin [139, 140, 155, 156]	Hydroxytyrosol acetate sulfate	–	Urine/plasma	Y	U	Y	U	Y	U	U	Y	U
Olive oil, (extra) virgin [136, 150, 151]	3-O-Methylhydroxytyrosol	–	Urine	Y	Y	U	U	Y	U	U	Y	U
Flaxseed/linseed oil [160–165]	α-Linolenic acid	HMDB0001388	Plasma/serum/erythrocytes/platelets	N	Y	U	Y	Y	U	U	Y	U
Rapeseed/canola oil [158, 175–178, 174, 179]	α-Linolenic acid	HMDB0001388	Plasma/platelets/breast milk	N	Y	Y	U	Y	U	U	Y	U
Sunflower oil [157, 173, 179, 178, 180]	Linoleic acid	HMDB0000673	Plasma/platelets/subcutaneous adipose tissue	N	U	U	U	Y	U	U	Y	U

*HMDB* human metabolome database, *N* no, *U* unknown, *Y* yes. Criteria: *C1*—*Plausibility*, Is the marker compound plausible as a specific BFI for the food or food group?; *C2*—*Dose response*, Is there a dose-response relationship at relevant intake levels of the targeted food?; *C3*—*Time response*, Is the biomarker kinetics described adequately to make a wise choice of sample type, frequency and time window?; *C3a*, *single dose*; *C3b*, *multiple doses*; *C4*, *Robustness*, Has the marker been shown to be robust after intake of complex meals reflecting the dietary habits of the targeted population?; *C5*, *Reliability*, Has the marker been shown to compare well with other markers or questionnaire data for the same food/food group?; *C6*, *Stability*, Is the marker chemically and biologically stable during biospecimen collection and storage, making measurements reliable and feasible?; *C7*, *Analytical performance*, Are analytical variability (CV%), accuracy, sensitivity and specificity known as adequate for at least one reported analytical method?; *C8*, *Reproducibility*, Has the analysis been successfully reproduced in another laboratory?

#### Olive oil

Olive oil is obtained from the fruits of the olive tree (*Olea europaea*) and its fatty acid constituent is predominantly oleic acid [C18:1(n-9)], and depending on type (refined, virgin, extra virgin oil), variable amounts of unsaponifiable fatty acids are present [182].

Several markers of (virgin) olive oil consumption have been identified in urine and blood, including tyrosol, hydroxytyrosol, and their metabolites. Dose-response relationships for the excretion of tyrosol and hydroxytyrosol in urine were observed in several studies using either a 1-day [136] or a 3-week crossover design [143, 144, 146–148]. Excretion of tyrosol and/or hydroxytyrosol was maintained when olive oil was included as an ingredient in the daily diet [80, 143, 145–147, 149–151]. For acute intakes of extra virgin olive oil, time-response relationships were described in plasma [183] and urine [135]. Most of the tyrosol, hydroxytyrosol, and metabolites were excreted within 6 h after administration of the dose. In a 4-week single-arm study, plasma hydroxytyrosol increased about fivefold after daily administration of 50 mL of extra virgin olive oil [149]. Also, (hydroxy)-tyrosol metabolites (3-O-methylhydroxytyrosol, homovanillic acid, homovanillic alcohol, and hydroxytyrosol sulfate) were identified in urine in a dose-dependent manner [136, 138, 140, 184]. After 3 weeks or more of daily ingestion of olive oils with varying phenolic content, these and other metabolites (hydroxytyrosol acetate sulfate, homovanillic alcohol sulfate, homovanillic acid sulfate, hydroxytyrosol sulfate, hydroxytyrosol acetate sulfate, and homovanillic acid glucuronide) increased in plasma [152, 154, 156] and urine [145, 149–151]. Ingestion of a single dose of olive oil with moderate to high phenolic content also resulted in an increase in the amount of metabolites in both urine [135] and plasma [137–139]. The increase in plasma metabolites occurred within 6 h after dosing. Hydroxytyrosol and its metabolites 3-O-methylhydroxytyrosol, hydroxytyrosol sulfate, and hydroxytyrosol acetate sulfate are probably specific for (extra) virgin olive oil [185]. Tyrosol is not only present in olives but also in wine. Homovanillic acid, homovanillyl alcohol, and their conjugated metabolites are also less specific: e.g., homovanillic acid is a dopamine metabolite occurring in human body fluids, while homovanillyl alcohol can be detected in honey as it is a constituent of the mandibular secretion of honeybees [185].

The effect of olive oil intake on change in the fatty acid profile in blood cells and plasma lipid fractions has also been studied both for acute intakes and during prolonged feeding. Acute changes in the amount of plasma C18:1(n-9) were observed within 3–4 h after a meal [133, 134]. Prolonged consumption of diets moderate to high in olive oil resulted in increases in the amount of oleic acid in plasma, plasma lipid fractions, and erythrocytes, as was shown in single-arm studies, crossover studies, and parallel studies that lasted 2–8 weeks [127–131, 141, 157]. A time response for repeated intakes of olive oil was also described [132, 141].

#### Flaxseed oil

Flaxseed oil or linseed oil is the oil obtained from the seed of the flax plant (*Linum usitatissimum L.*) and is known for its considerable amounts (> 50% of total fat) of ALA. Parallel or crossover feeding trials, lasting 2–12 weeks, with flaxseed oil in the daily diet showed increased incorporation of ALA in platelets and erythrocyte membranes and elevated levels in plasma lipid fractions [157, 159–171, 173]. A limited number of studies described a time-related increase [164, 166, 167] and a dose-dependent change [163, 169] in the biomedia. In several of these studies, changes in the level of elongation and desaturation products (stearidonic acid -C18:4(n-3)-, eicosatetraenoic -C20:4(n-3)-, EPA, and DHA) were also observed depending on the duration of the feeding.

#### Rapeseed (canola) oil

Oils produced from Brassica oilseeds are very low in erucic acid nowadays (C22:1 n-9), thanks to improvements in plant-breeding programs to grow low erucic acid cultivars [186]. The majority of fatty acids in rapeseed/canola oil are MUFAs, mainly oleic acid. The PUFA fraction consists of variable amounts of LA and ALA. The amount of ALA is much lower in rapeseed oil than in flaxseed oil, but the human consumption of rapeseed, either direct or as part of edible fats and other manufactured food, is higher. Biomarkers of the intake of rapeseed oil have focused on ALA. In several crossover studies ranging from 2.5 to 6 weeks in duration, levels of ALA in plasma lipid fractions and blood platelets increased after consumption of diets with increased levels of ALA from rapeseed or canola oil [157, 158, 177–179]. A dose-dependent increase was observed in one study [163]. Sampling the breastmilk of lactating women from 6 to 24 h up to 7 days after a dose of 40 g of canola oil revealed significantly increased amounts of ALA in the breast milk within 10 h [174].

#### Sunflower oil

Oil of the seeds of the sunflower (*Helianthus annuus L.*) is nutritionally valued by its high amounts of LA. Global consumption of sunflower oil ranks fourth after palm oil, rapeseed oil, and soybean oil [7]. Only high-oleic sunflower oil (HOSO) was known until a few decades ago. Newer sunflower hybrids yielding oils with high oleic acid content became available on the market more recently [187]. Crossover or parallel feeding studies ranging from 2.5 to 8 weeks with sunflower oil as a discerned source of fat in the diet showed increased levels of linoleic acid in plasma lipid fractions, platelets, and subcutaneous adipose tissue at the end of the intervention [157, 173, 178–180, 188]. For sunflower oil with a high oleic acid content, increased amounts of oleic acid in plasma lipid fractions and erythrocytes were observed after 3–5 weeks of feeding [131, 188–192]. To the best of our knowledge, acute- or repeated-intake time-response relationships have not been described for sunflower oil.

#### Other oils

A limited number of studies were found regarding other common oils such as safflower oil, corn oil, coconut oil, and soybean oil. These studies show that after prolonged feeding (of several weeks or longer), plasma/serum lipid fractions emerge as a potential putative biomarker [112, 158, 174, 192–199]. Data from these studies showed that in general, increasing the amounts of dietary fatty acids increase the level of fatty acids in blood lipid fractions, cell membranes, and adipose tissue. This is in line with the work of Hodson et al. [200], who reviewed the fatty acid composition of biological specimens as a biomarker of dietary intake. Fatty acids in biological specimens not synthesized endogenously [essential (n-6) and (n-3) fatty acids] correlate well with the intake of vegetable oils high in these fatty acids. The response, therefore, is specific for the fatty acid but not for the vegetable oil consumed. An inconvenient factor in studying fatty acids as biomarkers is that an increase in the level of one fatty acid inevitably leads to a decrease in the level of one or more other fatty acids. Furthermore, oils high in essential fatty acids, such as C18:3 (n-3) in flaxseed oil, generally increase incorporation and elevate the level of their fatty acid elongation products such as EPA and DHA. These observations were not taken into account in this review since the level of distinctiveness of such putative biomarkers progressively diminishes when other foods and food groups have similar components and are part of the same (endogenous) biochemical pathways.

## Conclusions

The most plausible candidate biomarkers for walnut intake are ALA, urolithins, and HIAA. Since these metabolites can also be detected after the intake of other foods, a combined model with all three metabolites could be a feasible solution for accurately monitoring walnut intake. In the case of almonds, α-tocopherol could potentially be a good candidate; however, here again a combination with other metabolites, such as catechin-derived metabolites, may improve the prediction of almond intake. For Brazil nuts, selenium may be a good candidate biomarker of intake, but it is a mineral widely distributed among other food sources. Thus, further untargeted metabolomics studies could be useful for finding additional candidate biomarkers with which to construct a multi-metabolite biomarker model. Similar needs exist for hazelnuts, macadamia nuts, peanuts, pecan nuts, and pistachios.

In regard to vegetable oils, several biomarkers of their intake have been described but none of them has been validated against other markers for the same food or food group. In the case of (virgin) olive oil, the most promising distinctive biomarker is hydroxytyrosol and its metabolites. In vegetable oils other than olive oil, fatty acids have been studied frequently, but these components lack sufficient distinctive sensitivity and specificity as biomarkers of the intake of vegetable oils. They represent a marker of the fatty acid itself rather than of the vegetable oil ingested. The analytical methods used in the reviewed literature can in general be considered sensitive and specific. Further discovery and validation studies are needed, which could focus on components in the unsaponifiable part of the oils.

Therefore, additional studies are necessary to discover new candidate BFIs, as well as to further evaluate the specificity, sensitivity, dose-response relationships, and reproducibility of these candidate biomarkers and to eventually validate them in other populations. For the discovery of new candidate BFIs, an untargeted metabolomics approach may be the most effective strategy, whereas for increasing the specificity of the evaluation of food consumption, this could be a combination of different metabolites.

## Additional file

Additional file 1: **Table S1.** Nonrelevant food intake biomarkers for nuts. **Table S2.** Nonrelevant food intake biomarkers for vegetable oils. (PDF 111 kb)

## Abbreviations

ALA: α-Linolenic acid
BFIs: Biomarkers of food intake
DHA: Docosahexaenoic acid
EA: Ellagic acid
EPA: Eicosapentaenoic acid
FFQs: Food frequency questionnaires
HIAA: Hydroxyindole-acetic acid
HOSO: High-linoleic sunflower oil
LA: Linoleic acid
MUFAs: Monounsaturated fatty acids
PUFAs: Polyunsaturated fatty acids
SFAs: Saturated fatty acids
