# Supplementary material for: Biomarkers of food intake for nuts and vegetable oils: an extensive literature search
Source: Genes Nutr. 2019 Mar 19;14:7. doi: 10.1186/s12263-019-0628-8 (PMC6423890; doi:10.1186/s12263-019-0628-8)
Supplement: Supplementary file 1 — Table S1. Nonrelevant food intake biomarkers for nuts. Table S2. Nonrelevant food intake biomarkers for vegetable oils. (PDF 111 kb) [file 12263_2019_628_MOESM1_ESM.pdf]

Supporting Information

Biomarkers of food intake biomarkers for nuts and vegetable oils: an extensive literature search

Mar Garcia-Aloy[1,2], Paul Hulshof[3], Sheila Estruel-Amades[1], Maryse C.J. Osté[4], Maria Lankinen[5], Johanna M. Geleijnse[3], Janette de Goede[3], Marynka Ulaszewska[6], Fulvio Mattivi[6,7], Stephan J.L. Bakker[4], Ursula Schwab[5], Cristina Andres-Lacueva*[1,2]

[1]Biomarkers and Nutrimetabolomics Laboratory, Department of Nutrition, Food Sciences and Gastronomy, XaRTA, INSA, Faculty of Pharmacy and Food Sciences, Campus Torribera, University of Barcelona, Barcelona, Spain

[2]CIBER de Fragilidad y Envejecimiento Saludable (CIBERFES), Instituto de Salud Carlos III, Barcelona, Spain

[3]Division of Human Nutrition and Health, Wageningen University, the Netherlands

[4]Department of Internal Medicine, University Medical Center Groningen, University of Groningen, Groningen, the Netherlands

[5] Institute of Public Health and Clinical Nutrition, University of Eastern Finland, Finland

[6]Department of Food Quality and Nutrition, Research Innovation Center, Fondazione Edmund Mach, Via Mach 1, 38010 San Michele all'Adige, TN, Italy

[7]Center Agriculture Food Environment, University of Trento, San Michele all'Adige, Italy.

Table S1. Nonrelevant food intake biomarkers for nuts

Compound / Metabolite [References]	HMDB ID	Dietary factor	Sample type	Reasons for exclusion
Linoleic acid [1–13]	HMDB0000673	Walnuts / Cashews / Mixed nuts	Plasma / Erythrocytes	Unspecific: common through different types of nuts and also more suitable FIB for some vegetable oils (i.e. safflower, sunflower, soybean and corn oils)
Eicosapentaenoic acid / Docosahexaenoic acid [2, 14, 15]	HMDB0001999 / HMDB0002183	Walnuts / Mixed nuts (walnuts and almonds)	Plasma / Serum	Unspecific: more suitable FIB for fatty fish
Oleic acid [5, 6, 16–23]	HMDB0000207	Walnuts / Almonds / Hazelnuts / Pecan nuts / Macadamia nuts / Cashews / Mixed nuts	Urine / Plasma / Serum / Erythrocytes	Unspecific: more suitable FIB for olive oil
Myristic acid / Stearic acid [1, 4, 24]	HMDB0000806 / HMDB0000827	Walnuts	Plasma / Serum / Erythrocytes	Unspecific: common to fat-rich foods
Arachidonic acid [25, 26]	HMDB0001043	Almonds / Nuts	Plasma	Unspecific: common to fat-rich foods
Palmitoleic acid [18, 20, 27, 28]	HMDB0003229	Macadamia nuts / Mixed nuts (walnuts, almonds, hazelnuts, macadamia nuts, pecans and cashews)	Serum / Plasma	Unspecific: common to fat-rich foods
Arachidic acid [18, 27]	HMDB0002212	Macadamia nuts	Plasma / Serum	Unspecific: common to fat-rich foods
Vaccenic acid / Eicosenoic acid [5, 23, 27]	HMDB0003231 / HMDB0002231	Macadamia nuts / Cashews	Plasma	Unspecific: common to fat-rich foods
Behenic acid [27, 29]	HMDB0000944	Macadamia nuts / Peanuts	Plasma	Unspecific: common to fat-rich foods
Lignoceric acid [29]	HMDB0002003	Peanuts	Plasma phospholipids	Unspecific: common to fat-rich foods
Suberic acid [21]	HMDB0000893	Mixed nuts (walnuts, almonds and hazelnuts)	Urine	Unspecific: common to fat-rich foods
Erucic acid / Osbond acid [20]	HMDB0002068 / –	Mixed nuts (walnuts, almonds, hazelnuts, macadamia nuts, pecans and cashews)	Serum	Unspecific: common to fat-rich foods
Dodecanedioic acid / Tridecadienoic/tridecynoic acid glucuronide / 10-Hydroxy-decene-4,6-diynoic acid sulfate [30–32]	HMDB0000623 / –	Walnuts / Mixed nuts (walnuts, almonds and hazelnuts)	Urine / Plasma	Unspecific: common to fat-rich foods
Monounsaturated fat [33, 34]	–	Almonds / Peanuts	Serum / Erythrocytes	Unspecific: common to fat-rich foods

Table S1 (continued)

Compound / Metabolite [References]	HMDB ID	Dietary factor	Sample type	Reasons for exclusion
(Epi)catechins and derived metabolites: (epi)catechin (and phase II metabolites), 4-hydroxy-5-(phenyl)-valeric acid sulfate, 4-hydroxy-5-(hydroxyphenyl)-valeric acid glucuronide/sulfate, 4-hydroxy-5-(dihydroxyphenyl)-valeric acid glucuronide/sulfate, 4-hydroxy-5-(methoxyphenyl)-valeric acid glucuronide, 5-(hydroxyphenyl)-γ-valerolactone glucuronide/sulfate, 5-(dihydroxyphenyl)-y-valerolactone (and phase II metabolites), 5-(trihydroxyphenyl)-γ-valerolactone glucuronide, 5-(hydroxymethoxyphenyl)-y-valerolactone glucuronide/sulfate [35–38]	–	Almonds	Urine / Plasma	Unspecific: common to flavan-3-ol-rich foods (mainly tea, cocoa, red wine)
Epi(gallo)catechin gallate [39, 40]	HMDB0037944 / HMDB0003153	Walnuts / Pecan nuts	Plasma	Unspecific: common to flavan-3-ol-rich foods
Naringenin (and phase II metabolites) / Isorhamnetin glucuronide / sulfate [35–38]	HMDB0002670 / HMDB0029209 / HMDB0041760 / HMDB0041761 / HMDB0041759 / HMDB0041757	Almonds	Urine Plasma	Unspecific
Enterolactone (and phase II metabolites) [31, 41]	HMDB0006101	Walnuts / Mixed nuts (walnuts and pecan nuts)	Urine Plasma	Unspecific: common to lignan-rich foods
p-Coumaryl alcohol glucuronide / sulfate / Pyrogallol sulfate [30]	HMDB0003654 / HMDB0060016 / HMDB0060018	Mixed nuts (walnuts, almonds and hazelnuts)	Urine	Unspecific
p-Cresol [21]	HMDB0001858	Mixed nuts (walnuts, almonds and hazelnuts)	Urine	Unspecific
4-Vinylphenol sulfate [42, 43]	HMDB0062775	Peanuts Nuts	Urine Serum	Unspecific
Ellagic acid / Dimethyl ellagic acid [44, 45]	HMDB0002899 / –	Walnuts	Feces / Prostate gland	Not usual sample type
Urolithins: urolithin D, urolithin M5, urolithin M6, urolithin M7 [44, 46]	HMDB0029219 / –	Walnuts	Feces	Not usual sample type

Table S1 (continued)

Compound / Metabolite [References]	HMDB ID	Dietary factor	Sample type	Reasons for exclusion
Phenolic acids: hydroxyhippuric acid, hydroxybenzoic acids (3-hydroxybenzoic acid, 4-hydroxybenzoic acid, 3,4-dihydroxybenzoic acid, vanillic acid, vanillic acid glucuronide), hydroxycinnamic acids (caffeic acid, coumaric acid, ferulic acid, ferulic acid glucuronide, 3-hydroxy-4-methoxycinnamic acid), hydroxyphenylacetic acids (2-(hydroxyphenyl)-acetic acid sulfate, 3-hydroxyphenylacetic acid, 2-(dihydroxyphenyl)-acetic acid glucuronide/sulfate/sulfoglucuronide, 2-(hydroxy-methoxy-phenyl)-acetic acid glucuronide, 4-hydroxy-3-methoxyphenylacetic acid, 4-methoxyphenylacetic acid, hydroxyphenylpropionic acids (3-(hydroxyphenyl)-propionic acid glucuronide, 3-(dihydroxyphenyl)-propionic acid sulfate, 3,4-dihydroxyphenylpropionic acid, phenylacetic acid [36, 38–40]	–	Walnuts / Almonds / Pecan nuts	Urine / Plasma	Unspecific: common to polyphenol-rich foods (cleavage product of gut microbiota)
Carotenes: α-Carotene, β-Carotene / Retinol [47, 48]	HMDB0003993 / HMDB0000561 / HMDB0000305 / HMDB0006217 / HMDB0006216 / HMDB0006221	Pistachios / Nuts	Plasma / Serum	Unspecific: vitamin A
Vitamin C [48, 49]	HMDB0000044	Nuts	Plasma	Unspecific: vitamin C
γ-Tocopherol [39, 40, 47, 50, 51]	HMDB0001492	Walnuts / Pecan nuts / Pistachios	Plasma / Serum	Unspecific: vitamin E
Vitamin B6 [11]	–	Walnuts	Plasma	Unspecific: vitamin B6
2-Pyridone-5-carboxymide + 1-methylnicotinamide [52]	–	Peanuts	Urine	Unspecific: vitamin B3 (derived metabolites)
Selenocystine [53]	HMDB0004122	Brazil nuts	Urine	Unspecific
Tryptophan betaine [42, 43]	–	Peanuts / Nuts	Urine / Serum	Unspecific
3-Indolecarboxylic acid glucuronide [31]	HMDB0013189	Walnuts	Urine	Unspecific
N-Acetylserotonin (and phase II metabolites) [30, 31]	HMDB0001238 / HMDB0060834	Walnuts / Mixed nuts (walnuts, almonds and hazelnuts)	Urine	Unspecific

Table S1 (continued)

Compound / Metabolite [References]	HMDB ID	Dietary factor	Sample type	Reasons for exclusion
Hypaphorine [54]	HMDB0061115	Peanuts	Human milk	Unspecific
Raffinose / Sucrose / Myoinositol [55]	HMDB0003213 / HMDB0000258 / HMDB0000211	Pistachios	Plasma	Unspecific
Lutein [47]	HMDB0003233	Pistachios	Serum	Unspecific
N-Acetylglutamine / Phenylacetylglutamine [21]	HMDB0006029 / HMDB0006344	Mixed nuts (walnuts, almonds and hazelnuts)	Urine	Unspecific

Table S2. Nonrelevant food intake biomarkers for vegetable oils

Compound / Metabolite [References]	HMDB ID	Dietary factor	Sample type	Reasons for exclusion
Tyrosol [56–65]	HMDB0004284	Olive oil: different phenolic content, extra virgin, virgin & refined	Urine / Plasma	Unspecific: it is also present in wine
Homovanillic acid + metabolites (glucuronide, sulfate) [66–70]	HMDB0000118 HMDB0011719	Olive oil: high phenolic content, extra virgin, virgin & refined	Urine / Plasma	Unspecific: is an endogenous metabolite
Homovanillyl alcohol + metabolites (glucuronide, sulfate) [65, 69–71]	HMDB0038925	Olive oil: different phenolic content, high phenolic content & virgin	Urine	Unspecific: it can also be detected in honey
Vanillin sulfate [72]	HMDB0041789	Olive oil: enriched & virgin	Plasma	Unspecific
Oleic acid [73–80]	HMDB0000207	Canola oil / Rapeseed oil / Sunflower oil, high oleic acid & high linoleic acid	Plasma / Erythrocytes / Platelets / Abdominal subcutaneous adipose tissue / Breastmilk	Unspecific
Linoleic acid [74, 77, 79, 81–86]	HMDB0000673	Sunflower oil: high oleic acid & high linoleic acid / Safflower oil / Corn oil / Soybean oil	Plasma / Serum / Erythrocytes / Platelets / Abdominal subcutaneous adipose tissue / Breastmilk	Unspecific
Lauric acid [82, 83]	HMDB0000638	Coconut oil	Plasma / Breastmilk	Unspecific
Gamma tocopherol [87, 88]	HMDB0001492	Corn oil	Plasma / Serum	Unspecific

References

1. Sabate J, Fraser GE, Burke K, Knutsen SF, Bennett H, Lindsted KD. Effects of walnuts on serum lipid levels and blood pressure in normal men. N Engl J Med. 1993;328:603–7.

2. Abbey M, Noakes M, Belling GB, Nestel PJ. Partial replacement of saturated fatty acids with almonds or walnuts lowers total plasma cholesterol and low-density-lipoprotein cholesterol. Am J Clin Nutr. 1994;59:995–9.

3. Burns-Whitmore B, Haddad E, Sabaté J, Rajaram S. Effects of supplementing n-3 fatty acid enriched eggs and walnuts on cardiovascular disease risk markers in healthy free-living lacto-ovo-vegetarians: a randomized, crossover, free-living intervention study. Nutr J. 2014;13:29.

4. Wu L, Piotrowski K, Rau T, Waldmann E, Broedl UC, Demmelmair H, et al. Walnut-enriched diet reduces fasting non-HDL-cholesterol and apolipoprotein B in healthy Caucasian subjects: a randomized controlled cross-over clinical trial. Metabolism. 2014;63:382–91.

5. Abdullah MMH, Cyr A, Lepine M-C, Labonte M-E, Couture P, Jones PJH, et al. Recommended dairy product intake modulates circulating fatty acid profile in healthy adults: a multi-centre cross-over study. Br J Nutr. 2015;113:435–44.

6. Chisholm a, Mann J, Skeaff M, Frampton C, Sutherland W, Duncan a, et al. A diet rich in walnuts favourably influences plasma fatty acid profile in moderately hyperlipidaemic subjects. Eur J Clin Nutr. 1998;52:12–6.

7. Zambón D, Sabaté J, Muñoz S, Campero B, Casals E, Merlos M, et al. Substituting walnuts for monounsaturated fat improves the serum lipid profile of hypercholesterolemic men and women. A randomized crossover trial. Ann Intern Med. 2000;132:538–46.

8. Almario RU, Vonghavaravat V, Wong R, Kasim-Karakas SE. Effects of walnut consumption on plasma fatty acids and lipoproteins in combined hyperlipidemia. Am J Clin Nutr. 2001;74:72–9.

9. Iwamoto M, Imaizumi K, Sato M, Hirooka Y, Sakai K, Takeshita A, et al. Serum lipid profiles in Japanese women and men during consumption of walnuts. Eur J Clin Nutr. 2002;56:629–37.

10. Rajaram S, Haddad EH, Mejia A, Sabate J. Walnuts and fatty fish influence different serum lipid fractions in normal to mildly hyperlipidemic individuals: a randomized controlled study. Am J Clin Nutr. 2009;89:1657S–1663S.

11. McKay DL, Chen C-YO, Yeum K-J, Matthan NR, Lichtenstein AH, Blumberg JB. Chronic and acute effects of walnuts on antioxidant capacity and nutritional status in humans: a randomized, cross-over pilot study. Nutr J. 2010;9:21.

12. Damasceno NRT, Perez-Heras A, Serra M, Cofan M, Sala-Vila A, Salas-Salvado J, et al. Crossover study of diets enriched with virgin olive oil, walnuts or almonds. Effects on lipids and other cardiovascular risk markers. Nutr Metab Cardiovasc Dis. 2011;21 Suppl 1:S14-20.

13. Fitschen PJ, Rolfhus KR, Winfrey MR, Allen BK, Manzy M, Maher MA. Cardiovascular effects of consumption of black versus English walnuts. J Med Food. 2011;14:890–8.

14. Marangoni F, Colombo C, Martiello A, Poli A, Paoletti R, Galli C. Levels of the n-3 fatty acid eicosapentaenoic acid in addition to those of alpha linolenic acid are significantly raised in blood lipids by the intake of four walnuts a day in humans. Nutr Metab Cardiovasc Dis. 2007;17:457–61.

15. Ortega FJ, Cardona-Alvarado MI, Mercader JM, Moreno-Navarrete JM, Moreno M, Sabater M, et al. Circulating profiling reveals the effect of a polyunsaturated fatty acid-enriched diet on common microRNAs. J Nutr Biochem. 2015;26:1095–101.

16. Rajaram S, Burke K, Connell B, Myint T, Sabate J. A monounsaturated fatty acid-rich pecan-enriched diet favorably alters the serum lipid profile of healthy men and women. J Nutr. 2001;131:2275–9.

17. Sabaté J, Haddad E, Tanzman JS, Jambazian P, Rajaram S. Serum lipid response to the graduated enrichment of a Step I diet with almonds: a randomized feeding trial. Am J Clin Nutr. 2003;77:1379–84.

18. Griel AE, Cao Y, Bagshaw DD, Cifelli AM, Holub B, Kris-Etherton PM. A macadamia nut-rich diet reduces total and LDL-cholesterol in mildly hypercholesterolemic men and women. J Nutr. 2008;138:761–7.

19. Orem A, Yucesan FB, Orem C, Akcan B, Kural BV, Alasalvar C, et al. Hazelnut-enriched diet improves cardiovascular risk biomarkers beyond a lipid-lowering effect in hypercholesterolemic subjects. J Clin Lipidol. 2013;7:123–31.

20. Nishi SK, Kendall CWC, Bazinet RP, Bashyam B, Ireland CA, Augustin LSA, et al. Nut consumption, serum fatty acid profile and estimated coronary heart disease risk in type 2 diabetes. Nutr Metab Cardiovasc Dis. 2014;24:845–52.

21. Vázquez-Fresno R, Llorach R, Urpi-Sarda M, Lupianez-Barbero A, Estruch R, Corella D, et al. Metabolomic pattern analysis after mediterranean diet intervention in a nondiabetic population: a 1- and 3-year follow-up in the PREDIMED study. J Proteome Res. 2015;14:531–40.

22. Deon V, Del Bo' C, Guaraldi F, Abello F, Belviso S, Porrini M, et al. Effect of hazelnut on serum lipid profile and fatty acid composition of erythrocyte phospholipids in children and adolescents with primary hyperlipidemia: A randomized controlled trial. Clin Nutr. 2017.

23. Mah E, Schulz JA, Kaden VN, Lawless AL, Rotor J, Mantilla LB, et al. Cashew consumption reduces total and LDL cholesterol: a randomized, crossover, controlled-feeding trial. Am J Clin Nutr. 2017;105:1070–8.

24. Campos Mondragón, M.G; Oliart Ros, R. M. ; Angulo Guerrero JO. Inflammatory markers in patients with metabolic syndrome after the intake of fatty acids n-3 and conjugated linoleic acid (CLA). Nutr Clínica y Dietética Hosp. 2013;33:7–17.

25. Chisholm A, Mc Auley K, Mann J, Williams S, Skeaff M. Cholesterol lowering effects

of nuts compared with a Canola oil enriched cereal of similar fat composition. Nutr Metab Cardiovasc Dis. 2005;15:284–92.

26. Kalgaonkar S, Almario RU, Gurusinghe D, Garamendi EM, Buchan W, Kim K, et al. Differential effects of walnuts vs almonds on improving metabolic and endocrine parameters in PCOS. Eur J Clin Nutr. 2011;65:386–93.

27. Garg ML, Blake RJ, Wills RBH. Macadamia nut consumption lowers plasma total and LDL cholesterol levels in hypercholesterolemic men. J Nutr. 2003;133:1060–3.

28. Hiraoka-Yamamoto J, Ikeda K, Negishi H, Mori M, Hirose A, Sawada S, et al. SERUM LIPID EFFECTS OF A MONOUNSATURATED (PALMITOLEIC) FATTY ACID-RICH DIET BASED ON MACADAMIA NUTS IN HEALTHY, YOUNG JAPANESE WOMEN. Clin Exp Pharmacol Physiol. 2004;31:S37–8.

29. Lemaitre RN, Fretts AM, Sitlani CM, Biggs ML, Mukamal K, King IB, et al. Plasma phospholipid very-long-chain saturated fatty acids and incident diabetes in older adults: the Cardiovascular Health Study. Am J Clin Nutr. 2015;101:1047–54.

30. Tulipani S, Llorach R, Jáuregui O, López-Uriarte P, Garcia-Aloy M, Bullo M, et al. Metabolomics unveils urinary changes in subjects with metabolic syndrome following 12-week nut consumption. J Proteome Res. 2011;10:5047–58.

31. Garcia-Aloy M, Llorach R, Urpi-Sarda M, Tulipani S, Estruch R, Martínez-González MA, et al. Novel Multimetabolite Prediction of Walnut Consumption by a Urinary Biomarker Model in a Free-Living Population: the PREDIMED Study. J Proteome Res. 2014;13:3476–83.

32. Mora-Cubillos X, Tulipani S, Garcia-Aloy M, Bulló M, Tinahones FJ, Andres-Lacueva C. Plasma metabolomic biomarkers of mixed nuts exposure inversely correlate with severity of metabolic syndrome. Mol Nutr Food Res. 2015;59.

33. Ghadimi Nouran M, Kimiagar M, Abadi A, Mirzazadeh M, Harrison G. Peanut consumption and cardiovascular risk. Public Health Nutr. 2010;13:1581–6.

34. Rajaram S, Connell KM, Sabaté J. Effect of almond-enriched high-monounsaturated fat diet on selected markers of inflammation: a randomised, controlled, crossover study. Br J Nutr. 2010;103:907–12.

35. Milbury PE, Chen C-Y, Blumberg JB. Temporal Effects of Almond Skin Polyphenols on Plasma Biomarkers of Redox Status. -- Milbury et al. 21 (5): A159 -- The FASEB Journal. FASEB J. 2007;21:352.6.

36. Urpi-Sarda M, Garrido I, Monagas M, Gómez-Cordovés C, Medina-Remón A, Andres-Lacueva C, et al. Profile of Plasma and Urine Metabolites after the Intake of Almond [Prunus dulcis (Mill.) D.A. Webb] Polyphenols in Humans. J Agric Food Chem. 2009;57:10134–42.

37. Garrido I, Urpi-Sarda M, Monagas M, Gómez-Cordovés C, Martín-Alvarez PJ, Llorach R, et al. Targeted analysis of conjugated and microbial-derived phenolic metabolites in human urine after consumption of an almond skin phenolic extract. J Nutr. 2010;140:1799–807.

38. Llorach R, Garrido I, Monagas M, Urpi-Sarda M, Tulipani S, Bartolome B, et al. Metabolomics study of human urinary metabolome modifications after intake of almond (Prunus dulcis (Mill.) D.A. Webb) skin polyphenols. J Proteome Res. 2010;9:5859–67.

39. Hudthagosol C, Haddad EH, McCarthy K, Wang P, Oda K, Sabate J. Pecans acutely increase plasma postprandial antioxidant capacity and catechins and decrease LDL oxidation in humans. J Nutr. 2011;141:56–62.

40. Haddad EH, Gaban-Chong N, Oda K, Sabaté J. Effect of a walnut meal on postprandial oxidative stress and antioxidants in healthy individuals. Nutr J. 2014;13:4.

41. Horner NK, Kristal AR, Prunty J, Skor HE, Potter JD, Lampe JW. Dietary Determinants of Plasma Enterolactone. Cancer Epidemiol Prev Biomarkers. 2002;11.

42. Guertin KA, Moore SC, Sampson JN, Huang W-Y, Xiao Q, Stolzenberg-Solomon RZ, et al. Metabolomics in nutritional epidemiology: identifying metabolites associated with diet and quantifying their potential to uncover diet-disease relations in populations. Am J Clin Nutr. 2014;100:208–17.

43. Playdon MC, Sampson JN, Cross AJ, Sinha R, Guertin KA, Moy KA, et al. Comparing metabolite profiles of habitual diet in serum and urine. Am J Clin Nutr. 2016;104:776–89.

44. Romo-Vaquero M, García-Villalba R, González-Sarrías A, Beltrán D, Tomás-Barberán FA, Espín JC, et al. Interindividual variability in the human metabolism of ellagic acid: Contribution of Gordonibacter to urolithin production. J Funct Foods. 2015;17:785–91.

45. González-Sarrías A, Giménez-Bastida JA, García-Conesa MT, Gómez-Sánchez MB, García-Talavera N V., Gil-Izquierdo A, et al. Occurrence of urolithins, gut microbiota ellagic acid metabolites and proliferation markers expression response in the human prostate gland upon consumption of walnuts and pomegranate juice. Mol Nutr Food Res. 2010;54:311–22.

46. Garcia-Villalba R, Espin JC, Tomas-Barberan FA, García-Villalba R, Espín JC, Tomás-Barberán FA. Chromatographic and spectroscopic characterization of urolithins for their determination in biological samples after the intake of foods containing ellagitannins and ellagic acid. J Chromatogr A. 2015;1428:162–75.

47. Kay CD, Gebauer SK, West SG, Kris-Etherton PM. Pistachios increase serum antioxidants and lower serum oxidized-LDL in hypercholesterolemic adults. J Nutr. 2010;140:1093–8.

48. Mohammadifard N, Salehi-Abargouei A, Salas-Salvado J, Guasch-Ferre M, Humphries K, Sarrafzadegan N. The effect of tree nut, peanut, and soy nut consumption on blood pressure: a systematic review and meta-analysis of randomized controlled clinical trials. Am J Clin Nutr. 2015;101:966–82.

49. Agebratt C, Strom E, Romu T, Dahlqvist-Leinhard O, Borga M, Leandersson P, et al. A Randomized Study of the Effects of Additional Fruit and Nuts Consumption on Hepatic Fat Content, Cardiovascular Risk Factors and Basal Metabolic Rate. PLoS One. 2016;11.

50. Ros E, Núñez I, Pérez-Heras A, Serra M, Gilabert R, Casals E, et al. A Walnut Diet Improves Endothelial Function in Hypercholesterolemic Subjects: A Randomized

Crossover Trial. Circulation. 2004;109:1609–14.

51. Hernandez-Alonso P, Salas-Salvado J, Baldrich-Mora M, Juanola-Falgarona M, Bullo M. Beneficial effect of pistachio consumption on glucose metabolism, insulin resistance, inflammation, and related metabolic risk markers: a randomized clinical trial. Diabetes Care. 2014;37:3098–105.

52. Seal AJ, Creeke PI, Dibari F, Cheung E, Kyroussis E, Semedo P, et al. Low and deficient niacin status and pellagra are endemic in postwar Angola. Am J Clin Nutr. 2007;85:218–24.

53. da Silva EG, Verola Mataveli LR, Zezzi Arruda MA. Speciation analysis of selenium in plankton, Brazil nut and human urine samples by HPLC–ICP-MS. Talanta. 2013;110:53–7.

54. Keller BO, Wu BTF, Li SSJ, Monga V, Innis SM. Hypaphorine is present in human milk in association with consumption of legumes. J Agric Food Chem. 2013;61:7654–60.

55. Nieman DC, Scherr J, Luo B, Meaney MP, Dreau D, Sha W, et al. Influence of pistachios on performance and exercise-induced inflammation, oxidative stress, immune dysfunction, and metabolite shifts in cyclists: a randomized, crossover trial. PLoS One. 2014;9:e113725.

56. Fitó M, Cladellas M, de la Torre R, Martí J, Muñoz D, Schröder H, et al. Anti-inflammatory effect of virgin olive oil in stable coronary disease patients: a randomized, crossover, controlled trial. Eur J Clin Nutr. 2008;62:570–4.

57. Covas M-I, de la Torre K, Farré-Albaladejo M, Kaikkonen J, Fitó M, López-Sabater C, et al. Postprandial LDL phenolic content and LDL oxidation are modulated by olive oil phenolic compounds in humans. Free Radic Biol Med. 2006;40:608–16.

58. Perona JS, Fitó M, Covas M-I, Garcia M, Ruiz-Gutierrez V. Olive oil phenols modulate the triacylglycerol molecular species of human very low-density lipoprotein. A randomized, crossover, controlled trial. Metabolism. 2011;60:893–9.

59. Machowetz A, Gruendel S, Garcia A, Harsch I, Covas M-I, Zunft H-J, et al. Effect of Olive Oil Consumption on Serum Resistin Concentrations in Healthy Men. Horm Metab Res. 2008;40:697–701.

60. Marrugat J, Covas M-I, Fitó M, Schröder H, Miró-Casas E, Gimeno E, et al. Effects of differing phenolic content in dietary olive oils on lipids and LDL oxidation. Eur J Nutr. 2004;43:140–7.

61. Gimeno E, de la Torre-Carbot K, Lamuela-Raventós RM, Castellote AI, Fitó M, de la Torre R, et al. Changes in the phenolic content of low density lipoprotein after olive oil consumption in men. A randomized crossover controlled trial. Br J Nutr. 2007;98:1243–50.

62. Weinbrenner T, Fitó M, Farré Albaladejo M, Saez GT, Rijken P, Tormos C, et al. Bioavailability of phenolic compounds from olive oil and oxidative/antioxidant status at postprandial state in healthy humans. Drugs Exp Clin Res. 2004;30:207–12.

63. Bonanome A, Pagnan A, Caruso D, Toia A, Xamin A, Fedeli E, et al. Evidence of postprandial absorption of olive oil phenols in humans. Nutr Metab Cardiovasc Dis.

2000;10:111–20.

64. Machowetz A, Poulsen HE, Gruendel S, Weimann A, Fitó M, Marrugat J, et al. Effect of olive oils on biomarkers of oxidative DNA stress in Northern and Southern Europeans. FASEB J. 2007;21:45–52.

65. Khymenets O, Farré M, Pujadas M, Ortiz E, Joglar J, Covas MI, et al. Direct analysis of glucuronidated metabolites of main olive oil phenols in human urine after dietary consumption of virgin olive oil. Food Chem. 2011;126:306–14.

66. Fernández-Ávila C, Montes R, Castellote AI, Chisaguano AM, Fitó M, Covas MI, et al. Fast determination of virgin olive oil phenolic metabolites in human high-density lipoproteins. Biomed Chromatogr. 2015;29:1035–41.

67. Oliveras-López M-J, Innocenti M, Martín Bermudo F, López-García de la Serrana H, Mulinacci N. Effect of extra virgin olive oil on glycaemia in healthy young subjects. Eur J Lipid Sci Technol. 2012;114:999–1006.

68. de la Torre-Carbot K, Chávez-Servín JL, Jaúregui O, Castellote AI, Lamuela-Raventós RM, Nurmi T, et al. Elevated Circulating LDL Phenol Levels in Men Who Consumed Virgin Rather Than Refined Olive Oil Are Associated with Less Oxidation of Plasma LDL. J Nutr. 2010;140:501–8.

69. Caruso D, Visioli F, Patelli R, Galli C, Galli G. Urinary excretion of olive oil phenols and their metabolites in humans. Metabolism. 2001;50:1426–8.

70. Serra A, Rubió L, Macià A, Valls R-M, Catalán Ú, de la Torre R, et al. Application of dried spot cards as a rapid sample treatment method for determining hydroxytyrosol metabolites in human urine samples. Comparison with microelution solid-phase extraction. Anal Bioanal Chem. 2013;405:9179–92.

71. Salvini S, Sera F, Caruso D, Giovannelli L, Visioli F, Saieva C, et al. Daily consumption of a high-phenol extra-virgin olive oil reduces oxidative DNA damage in postmenopausal women. Br J Nutr. 2006;95:742–51.

72. Suárez M, Valls RM, Romero M-P, Macià A, Fernández S, Giralt M, et al. Bioavailability of phenols from a phenol-enriched olive oil. Br J Nutr. 2011;106:1691–701.

73. Corner EJ, Bruce VM, McDonald BE. Accumulation of eicosapentaenoic acid in plasma phospholipids of subjects fed canola oil. Lipids. 1990;25:598–601.

74. Larsen LF, Jespersen J, Marckmann P. Are olive oil diets antithrombotic? Diets enriched with olive, rapeseed, or sunflower oil affect postprandial factor VII differently. Am J Clin Nutr. 1999;70:976–82.

75. Cater NB, Heller HJ, Denke MA. Comparison of the effects of medium-chain triacylglycerols, palm oil, and high oleic acid sunflower oil on plasma triacylglycerol fatty acids and lipid and lipoprotein concentrations in humans. Am J Clin Nutr. 1997;65:41–5.

76. Denke MA, Grundy SM. Comparison of effects of lauric acid and palmitic acid on plasma lipids and lipoproteins. Am J Clin Nutr. 1992;56:895–8.

77. Wardlaw GM, Snook JT. Effect of diets high in butter, corn oil, or high-oleic acid

sunflower oil on serum lipids and apolipoproteins in men. Am J Clin Nutr. 1990;51:815–21.

78. Choudhury N, Truswell S, McNeil Y. Comparison of Plasma Lipids and Vitamin E in Young and Middle-Aged Subjects on Potato Crisps Fried in Palmolein and Highly Oleic Sunflower Oil. Ann Nutr Metab. 1997;41:137–48.

79. Solà R, La Ville AE, Richard JL, Motta C, Bargalló MT, Girona J, et al. Oleic acid rich diet protects against the oxidative modification of high density lipoprotein. Free Radic Biol Med. 1997;22:1037–45.

80. Ruíz-Gutiérrez V, Muriana FJ, Guerrero A, Cert AM, Villar J. Plasma lipids, erythrocyte membrane lipids and blood pressure of hypertensive women after ingestion of dietary oleic acid from two different sources. J Hypertens. 1996;14:1483–90.

81. Sanders K, Johnson L, O'Dea K, Sinclair AJ. The effect of dietary fat level and quality on plasma lipoprotein lipids and plasma fatty acids in normocholesterolemic subjects. Lipids. 1994;29:129–38.

82. Francois CA, Connor SL, Wander RC, Connor WE. Acute effects of dietary fatty acids on the fatty acids of human milk. Am J Clin Nutr. 1998;67:301–8.

83. Cox C, Mann J, Sutherland W, Chisholm A, Skeaff M. Effects of coconut oil, butter, and safflower oil on lipids and lipoproteins in persons with moderately elevated cholesterol levels. J Lipid Res. 1995;36:1787–95.

84. Schwab US, Vogel S, Lammi-Keefe CJ, Ordovas JM, Schaefer EJ, Li Z, et al. Varying Dietary Fat Type of Reduced-Fat Diets Has Little Effect on the Susceptibility of LDL to Oxidative Modification in Moderately Hypercholesterolemic Subjects. J Nutr. 1998;128:1703–9.

85. Lee TC, Ivester P, Hester AG, Sergeant S, Case L, Morgan T, et al. The impact of polyunsaturated fatty acid-based dietary supplements on disease biomarkers in a metabolic syndrome/diabetes population. Lipids Health Dis. 2014;13:196.

86. Uusitalo U, Feskens EJ, Tuomilehto J, Dowse G, Haw U, Fareed D, et al. Fall in total cholesterol concentration over five years in association with changes in fatty acid composition of cooking oil in Mauritius: cross sectional survey. BMJ. 1996;313:1044–6.

87. Tomasch R, Wagner K-H, Elmadfa I. Antioxidative Power of Plant Oils in Humans: The Influence of α- and γ-Tocopherol. Ann Nutr Metab. 2001;45:110–5.

88. Lemcke-Norojärvi M, Kamal-Eldin A, Appelqvist L-A, Dimberg LH, Öhrvall M, Vessby B. Corn and Sesame Oils Increase Serum γ-Tocopherol Concentrations in Healthy Swedish Women. J Nutr. 2001;131:1195–201.
